# Clinical prediction model based on 18F-FDG PET/CT plus contrast-enhanced MRI for axillary lymph node macrometastasis

**DOI:** 10.3389/fonc.2022.989650

**Published:** 2022-09-13

**Authors:** Shun Kawaguchi, Nobuko Tamura, Kiyo Tanaka, Yoko Kobayashi, Junichiro Sato, Keiichi Kinowaki, Masato Shiiba, Makiko Ishihara, Hidetaka Kawabata

**Affiliations:** ^1^Breast and Endocrine Surgery, Toranomon Hospital, Tokyo, Japan; ^2^Pathology, Toranomon Hospital, Tokyo, Japan; ^3^Diagnostic Imaging Center, Toranomon Hospital, Tokyo, Japan

**Keywords:** breast cancer, PET/CT, MRI, PET/MRI, axillary lymph node metastasis, macrometastasis, micrometastasis, logistic regression

## Abstract

**Purpose:**

Positron emission tomography/computed tomography (PET/CT) and magnetic resonance imaging (MRI) are useful for detecting axillary lymph node (ALN) metastasis in invasive ductal breast cancer (IDC); however, there is limited clinical evidence to demonstrate the effectiveness of the combination of PET/CT plus MRI. Further axillary surgery is not recommended against ALN micrometastasis (lesion ≤2 mm) seen in sentinel lymph nodes, especially for patients who received proper adjuvant therapy. We aimed to evaluate the efficacy of a prediction model based on PET/CT plus MRI for ALN macrometastasis (lesion >2 mm) and explore the possibility of risk stratification of patients using the preoperative PET/CT plus MRI and biopsy findings.

**Materials and methods:**

We retrospectively investigated 361 female patients (370 axillae; mean age, 56 years ± 12 [standard deviation]) who underwent surgery for primary IDC at a single center between April 2017 and March 2020. We constructed a prediction model with logistic regression. Patients were divided into low-risk and high-risk groups using a simple integer risk score, and the false negative rate for ALN macrometastasis was calculated to assess the validity. Internal validation was also achieved using a 5-fold cross-validation.

**Results:**

The PET/CT plus MRI model included five predictor variables: maximum standardized uptake value of primary tumor and ALN, primary tumor size, ALN cortical thickness, and histological grade. In the derivation (296 axillae) and validation (74 axillae) cohorts, 54% and 61% of patients, respectively, were classified as low-risk, with a false-negative rate of 11%. Five-fold cross-validation yielded an accuracy of 0.875.

**Conclusions:**

Our findings demonstrate the validity of the PET/CT plus MRI prediction model for ALN macrometastases. This model may aid the preoperative identification of low-risk patients for ALN macrometastasis and provide helpful information for PET/MRI interpretation.

## 1 Introduction

Breast cancer is the leading cause of cancer mortality among women in over 100 countries (1). Breast cancer metastasis is preceded by colonization of tumor cells circulating in the blood and lymphatics after their detachment from the primary tumor (2). Precise preoperative evaluation of the axillary status is important in clinical settings since axillary lymph node (ALN) metastasis is a strong poor prognostic factor and largely influences the primary breast cancer management (3). ALN dissection (ALND) is performed to assess the ALN status and improve local treatment (4); however, based on the results of the American College of Surgeons Oncology Group (ACOSOG) Z0011 trial, ALND can be avoided for patients who underwent lumpectomy for T1 or T2 breast cancer with 1 or 2 sentinel lymph nodes (SLNs) containing metastases, when they receive proper adjuvant radiotherapy in the absence of a history of neoadjuvant chemotherapy (5). ALN metastasis >2 mm is considered to be a macrometastasis (6), and the evidence for ALND omission for patients with more than 3 positive SLNs is insufficient for safety assessment. Another randomized clinical trial (International Breast Cancer Study Group 23-01) demonstrated that ALND for ALN micrometastases (≤2 mm) did not improve the overall survival (7). Although evidence for patients who underwent mastectomy is limited, because approximately 90% of such patients were treated by lumpectomy in the International Breast Cancer Study Group 23-01 trial, further axillary surgery is not recommended for patients with micrometastasis in the SLNs who underwent mastectomy by the 2022 National Comprehensive Cancer Network guideline (8). In the RxPONDER trial (9) of chemotherapy in women with hormone receptor-positive and node-positive disease including ALN micrometastasis, premenopausal women benefited from chemotherapy.

ALN can be evaluated through various imaging methods, with unique advantages and disadvantages, including magnetic resonance imaging (MRI), positron emission tomography/computed tomography (PET/CT), PET/MRI, and ultrasound (US). PET/CT showed low sensitivity of 59–69%, with poor spatial resolution, but high specificity of 90–95% (10). Comparatively, MRI has a higher sensitivity but lower specificity (77%, 95% confidence interval, [CI] 75–80%; 90%, 95% CI, 89–91%, respectively), and an average negative predictive value of 80% (11, 12). PET/MRI is designed to combine the metabolic data and high-contrast morphological features of PET/CT and MRI, respectively; however, it is inaccessible due to its high cost and insufficient evidence (13). US showed wide fluctuations in sensitivity and specificity, approximately 50–90%, since its quality largely depended on the operator (14). Identifying ALN metastases solely by imaging modalities is suboptimal; therefore, invasive axillary staging methods, such as sentinel lymph node biopsy (SLNB), are often performed, even for breast cancers with low malignant potential. This has previously been reported to cause shoulder and arm morbidities following SLNB, leading to loss of mobility, sensory disorders, and pain, impairing patient quality of life (15).

This study aimed to establish a clinical prediction model for ALN macrometastasis of primary invasive ductal carcinoma (IDC) utilizing the preoperative radiologic imaging and biopsy findings. We also developed a simple integer risk score and assessed its predictive ability for ALN macrometastasis using 5-fold cross-validation for internal validation and investigated the association of PET/CT plus MRI findings with ALN micrometastasis.

## 2 Materials and methods

### 2.1 Study design

This retrospective, single-center cohort study included patients with newly diagnosed primary IDC in Minato City, Tokyo, Japan. Both PET/CT and MRI were routinely used to preoperatively assess the nodal staging and tumor extension of breast cancer at our institution. The study protocol was approved by the respective ethics committee and the requirement for informed consent was waived owing to the retrospective and anonymous nature of the data. All participants had histologically proven IDC before surgery through core-needle biopsy (CNB) or vacuum-assisted biopsy (VAB). We performed partial or total mastectomy, SLNB, and/or ALND for the patients. Pathological examination of the resected ALNs was performed using SLNB alone, SLNB and ALND, or ALND alone. Upon comparing the pathologic information with the imaging findings, we proposed a clinical prediction model for ALN macrometastasis using logistic regression and reviewed its prediction ability in the internal validation group using a risk scoring system. Additionally, we performed a 5-fold cross-validation.

### 2.2 Patients

**Figure 1:**
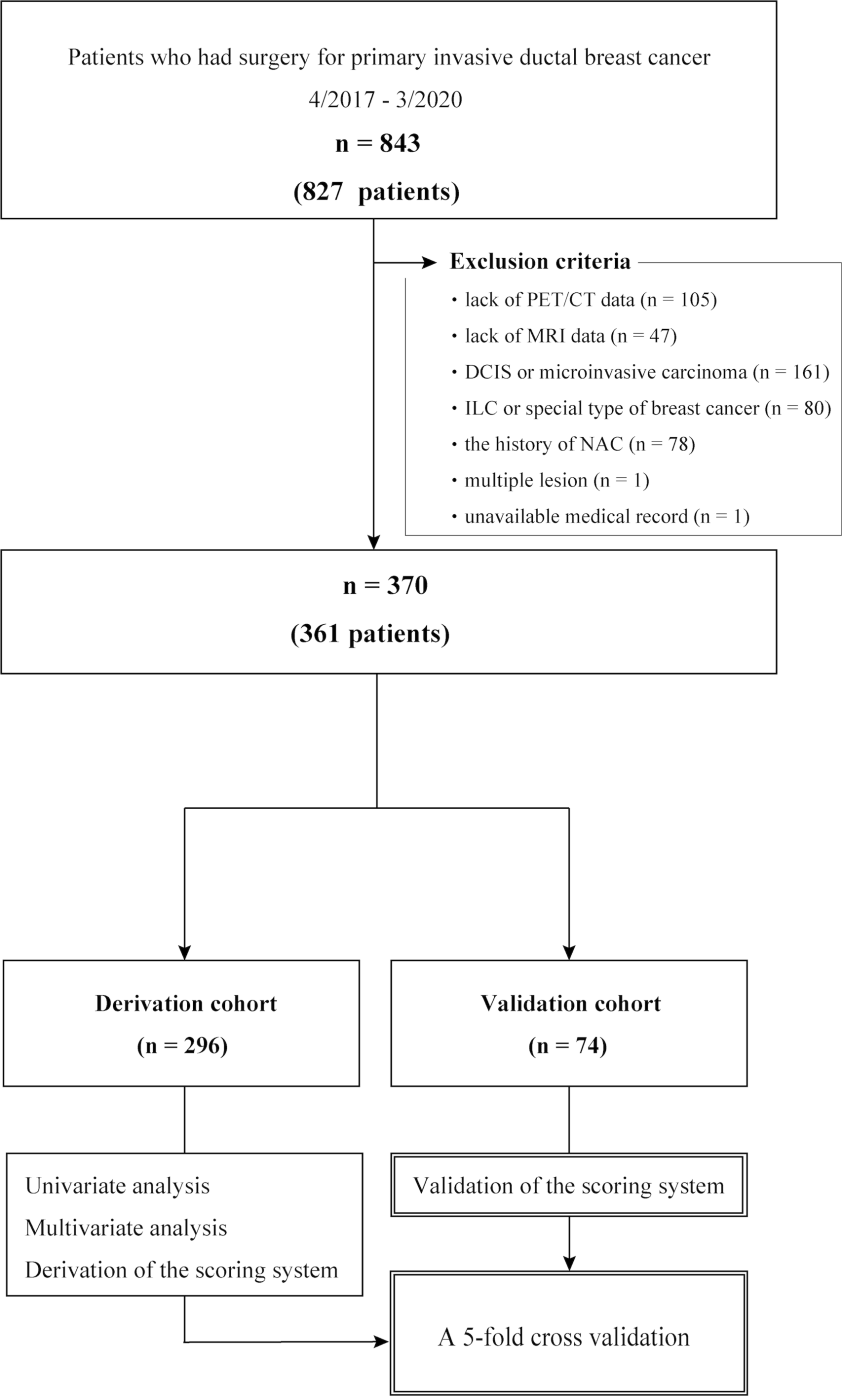
Cohort selection flow chart. DCIS, ductal carcinoma *in situ*; ILC, invasive lobular carcinoma; NAC, neoadjuvant chemotherapy.

Overall, 827 patients (843 axillae) that underwent surgical resection of primary IDC with SLNB and/or ALND between April 2017 and March 2020 were eligible for this study. The exclusion criteria included the absence of PET/CT data (n = 105) and MRI data (n = 47), diagnosis of ductal carcinoma *in situ* or microinvasive carcinoma (n = 161), diagnosis of invasive lobular carcinoma or a special type of breast cancer (n = 80), history of neoadjuvant chemotherapy (n = 78), multiple lesions (n = 1), and unavailable medical records (n = 1). No case was excluded based on age, presence of other cancers, or other comorbidities. Overall, 361 patients (370 axillae) were included in this analysis; nine patients had synchronous bilateral IDC (**Figure 1**). Tumor and nodal stages were retrospectively allocated according to the TNM staging system proposed by the American Joint Committee on Cancer, 8^th^ edition (6).

### 2.3 18F-Fluoro-2-deoxy-D-glucose PET/CT protocol and image analysis

18F-Fluoro-2-deoxy-D-glucose PET/CT scans were obtained using a BiographTM mCT Flow PET/CT system (Siemens Molecular Imaging, Hoffman Estates, IL, USA). The detailed PET/CT Protocol are provided in Supplementary Data (online). After image reconstruction, the SUV was measured for all primary breast cancer lesions and enlarged ALNs and represented as pSUVmax and LN-SUVmax, respectively. PET/CT images were reviewed in consensus by two experienced radiologists [M.I. and M.S. (radiologists with >20 and 19 years of experience, respectively)].

### 2.4 MRI protocol and image analysis

**Figure 2:**
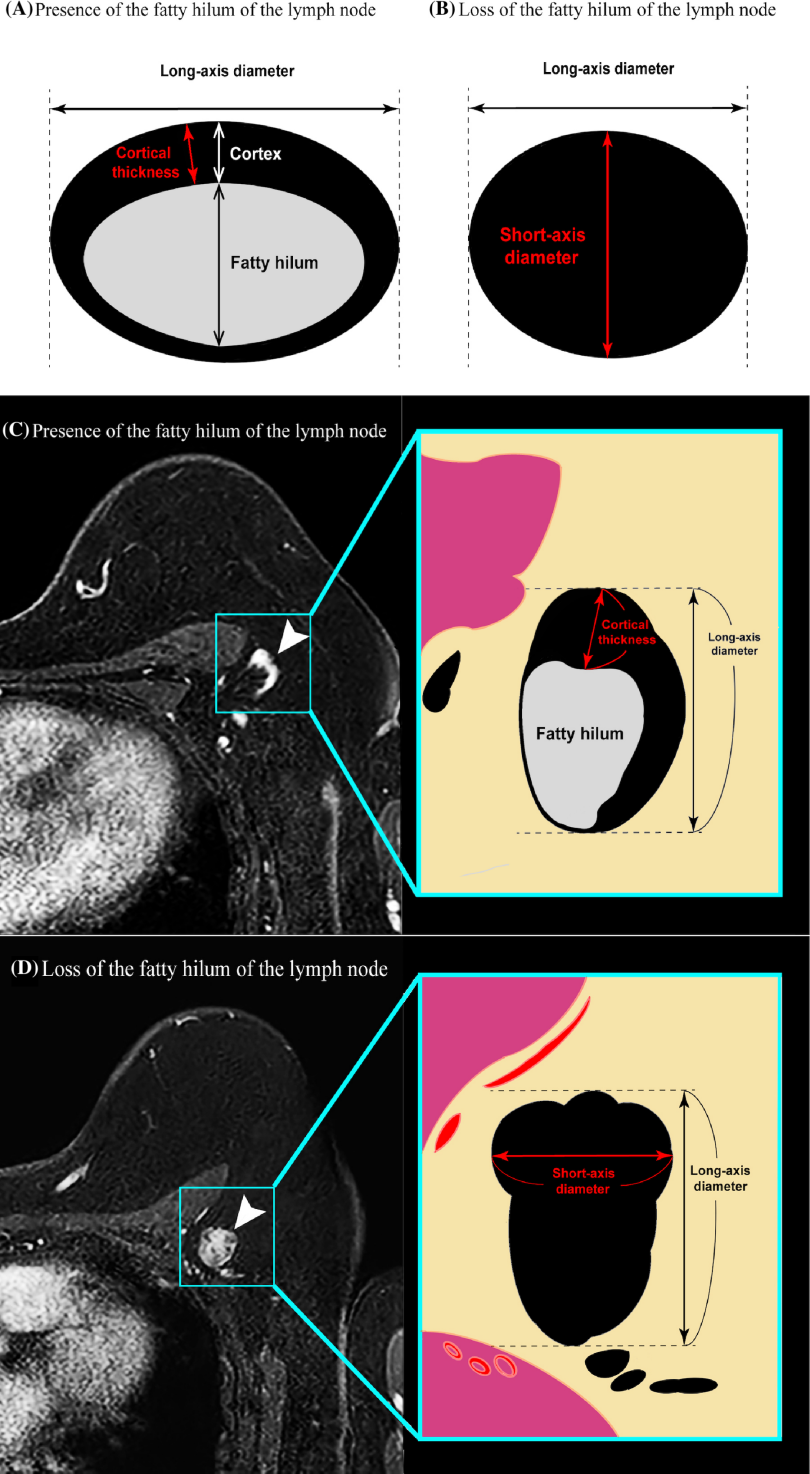
Diagrams illustrating the principle of the cortical thickness measurement method in a patient **(A)** in whom the hilum of the lymph node can be clearly identified versus a patient **(B)** in whom the hilum of the lymph node cannot be identified. In the former case, maximum cortical thickness was measured. In the latter case, the short-axis diameter was substituted for the cortical thickness. **(C, D)** Transverse contrast-enhanced T1-weighted MR images showing examples of the measurement of axillary lymph node cortical thickness in a patient **(C)** in whom the hilum of the lymph node can be clearly identified versus a patient **(D)** in whom the hilum of the lymph node cannot be clearly identified.

Dynamic contrast-enhanced MRI was performed using a 3.0-Tesla (T) MRI scanner (Ingenia, Philips Healthcare, Best, The Netherlands) with 7 channel breast coils. The detailed MRI protocol and image analysis are provided in **Supplementary Data** (online). The cortical thickness and long-axis diameter of all detectable ALNs on the ipsilateral side were evaluated on the horizontal dimension, and the data of the lymph node that had the largest cortical thickness were employed (**Figures 2A, C**). However, when loss of the fatty hilum was observed, the lymph node presenting such image findings was selected, and the short-axis diameter was measured as the cortical thickness (**Figures 2B, D**).

### 2.5 SLNB and ALND

SLNB was performed using isotopic and color dying methods with phytate sodium hydrate and technetium phytic acid, and indigo carmine, respectively. The SLNs were frozen sectioned at a thickness of 2 mm and examined by an expert pathologist using hematoxylin and eosin staining. During the intraoperative consultation of the SLN, we performed ALN sampling or ALND to level I/II according to the guideline (16). When ALN metastases of the SLN had not been detected until the final diagnosis, the secondary ALND or further adjuvant treatment was performed based on the case corresponding to the ACOSOG Z0011 trial (5).

### 2.6 Pathological evaluation

Tissue specimens of the primary IDC were obtained from preoperative CNB or VAB and surgical treatment. Tumor grading was based on the Nottingham grading system (17). Immunohistochemistry analyses were performed using a BenchMark GX automated staining instrument (Ventana Medical Systems, Inc., USA) for estrogen receptor (ER), progesterone receptor (PR), human epidermal growth factor receptor 2 (HER2), and Ki-67 using monoclonal rabbit anti-human antibodies against ER, PR, HER2 and Ki-67 (clone SP1; clone 1E2; clone 4B5 and clone 30-9, respectively), according to the manufacturer’s recommendations. The detailed Pathological Evaluation is in Supplementary Data (online).

### 2.7 Statistical analysis

Using a split-sample design, 80% of the patients were randomly selected as the derivation cohort (n = 296), while the remaining 20% were assigned to the internal validation cohort (n = 74) for risk scoring. In the derivation cohort, an optimal cutoff value of each variable for ALN macrometastasis was identified using a receiver-operating characteristic curve analysis. Univariate analysis using Fisher’s exact test was performed for all candidate variables, and those with a P value less than 0.20 were added to the logistic regression analysis. The β coefficients and odds ratios of the explanatory variables were estimated using logistic regression, and the β coefficients were multiplied by three and rounded to the nearest integer to construct a weighted scoring system. In the PET/CT plus MRI model, the cortical thickness of the lymph node was employed rather than the long-axis diameter in reference to the previous report (18), which described cortical thickness as an independent factor of ALN metastasis. Consequently, the cutoff value for the scoring system was determined; more than half of the patients scored less than the cutoff value, with a higher false-negative rate. The patients were divided into low-risk and high-risk groups according to the total score. Diagnostic accuracy was evaluated in the validation cohort and model discrimination was assessed using the c-index, which is the area under the receiver operator curve. Additionally, we used 5-fold cross-validation to assess model overfitting. Finally, multivariate logistic regression analysis following univariate analysis using Fisher’s exact test was performed for all candidate variables for ALN micrometastasis, and prognostic accuracy of ALN micrometastasis was assessed using the c-index. Data are expressed as the mean ± standard deviation. Continuous variables were evaluated using Student’s t-test analysis of variance. A p value of less than 0.05 was accepted as the level of significance for all the analyses. R: A language and environment for statistical computing version, 4.0.3 (R Foundation for Statistical Computing, Vienna, Austria) was used.

## 3 Results

### 3.1 Flow chart and cohort selection

Of the 361 female patients with breast cancer (370 axillae; mean age, 56 years ± 12 [standard deviation]), 60 axillae (16%) had ALN macrometastasis. The baseline characteristics were similar between the derivation (n = 296) and validation (n = 74) cohorts (Supplementary Data, **Supplementary Table 1**). Among the initial 827 participants (840 axillae), 466 (470 axillae) were excluded from analysis (**Figure 1**).

### 3.2 Patient characteristics

**Figure 3:**
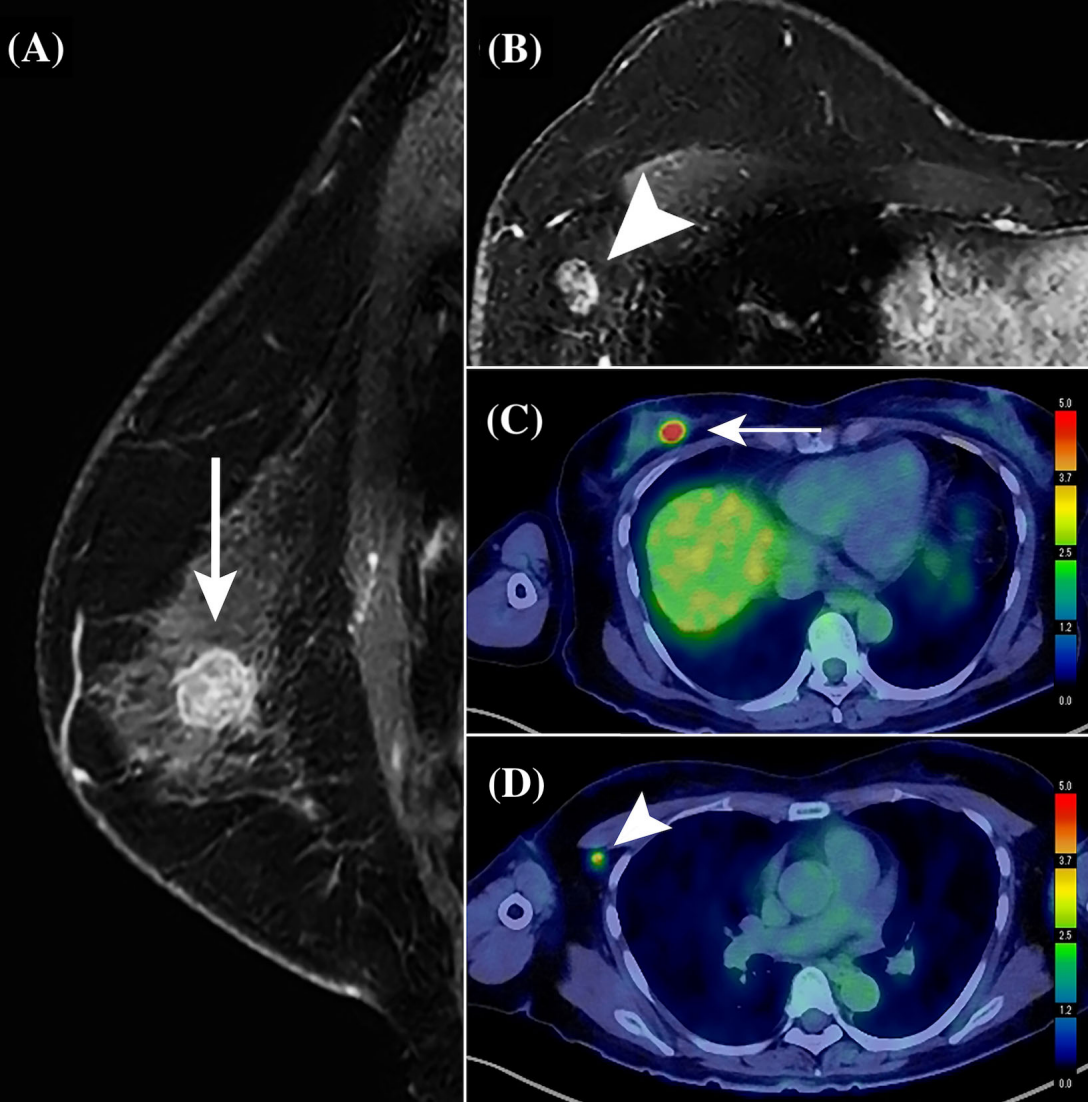
Representative contrast-enhanced MR and PET/CT images of a 59-year-old woman with primary breast cancer (pT1cN1aM0, pStage IA; invasive ductal carcinoma; Luminal A-like type; histological grade, II). On using the perioperative risk scoring system for axillary lymph node (ALN) macrometastasis, the patient with a total score of 15 was categorized into the high-risk group. A solitary ALN macrometastasis of 8 mm was detected by sentinel lymph node biopsy followed by ALN dissection. **(A)** Sagittal contrast-enhanced T1-weighted MR image shows a primary invasive ductal breast cancer in the upper and inner quadrant of the right breast, a 15-mm round mass (white arrow), with heterogenous enhancement pattern. **(B)** Transverse T1-weighted MR image showing an enlarged ALN exhibiting no fatty hilum, with a cortical thickness of 9 mm (white arrow). **(C)** PET/CT shows an abnormal FDG accumulation (white arrow) in the right breast mass, with a maximum standardized uptake value (SUVmax) of 9.45. **(D)** PET/CT also shows marked FDG accumulation in an enlarged ALN (white arrow), with an SUVmax of 4.96.

Patient characteristics were compared between the two groups with (n = 245) or without ALN macrometastasis (n = 51) in the derivation cohort (**Table 1**). The average primary tumor SUVmax, lymph node SUVmax, MRI tumor size, lymph node long-axis diameter, and cortical thickness of the patients who had ALN macrometastasis were significantly larger than those of the patients with non-metastatic ALN (**Figure 3**, p < 0.05). In addition to the PET/CT and MRI findings, a high histological grade (HG) evaluated by CNB or VAB was significantly associated with positive ALN macrometastasis (p < 0.001).

**Table 1:** Patients’ characteristics with or without axillary lymph node macrometastasis in the derivation cohort.

Characteristics	Macrometastasis (-) (n = 245)	Macrometastasis (+) (n = 51)	p value
Age, years*	55.9 ± 12.0	55.5 ± 12.1	0.84
Histological grade			<0.001
I	90 (37)	6 (12)	
II	132 (54)	39 (76)	
III	23 (9)	6 (12)	
Nuclear grade			0.56
1	130 (53)	23 (45)	
2	88 (36)	22 (43)	
3	27 (11)	6 (12)	
Ki-67 grade			0.36
<20%	133 (54)	24 (47)	
≥20%	112 (46)	27 (53)	
Lymphovascular invasion			<0.001
Absence	191 (78)	9 (18)	
Presence	54 (22)	42 (82)	
Tumor size in pathology* (mm)	12.3 ± 6.6	20.8 ± 11.5	<0.001
Molecular subtypes			0.37
Luminal A-like (Ki-67 <20%)	117 (48)	18 (35)	
Luminal B-like (Ki-67 ≥20%)	91 (37)	23 (45)	
Luminal-HER2	16 (7)	5 (10)	
Pure HER2	8 (3)	3 (6)	
Triple-negative	13 (5)	2 (4)	
Nodal FDG uptake finding			<0.001
Negative	225 (92)	21 (41)	
Positive	20 (8)	30 (59)	
Primary tumor SUVmax*	6.8 ± 6.1	9.1 ± 7.0	0.015
Lymph node SUVmax*	2.6 ± 2.1	5.2 ± 4.6	0.028
MRI tumor size* (mm)	14.9 ± 7.8	22.6 ± 10.5	<0.001
MRI lymph node size* (mm)			
Long-axis diameter	7.0 ± 2.9	10.8 ± 5.3	<0.001
Cortical thickness	3.9 ± 1.5	6.4 ± 3.6	<0.001

FDG, 18F-fluoro-2-deoxy-D-glucose; SUVmax, maximum standardized uptake value; MRI, magnetic resonance imaging.* Data presented as means ± standard deviation.

### 3.3 Model construction and validation for ALN macrometastasis

**Table 2** displays the p-values of each predictor for ALN macrometastasis using univariate analysis, and the β coefficients, odds ratio, and p value of each multivariate model according to the imaging modalities. Univariate analysis demonstrated that HG (II and III vs. I), lymph node SUVmax (≥1.2 vs. <1.2), MRI tumor size (≥19 mm vs. <19 mm), lymph node long-axis diameter (≥8 mm vs. <8 mm), and cortical thickness (≥5 mm vs. <5 mm) were significantly associated with the presence of ALN macrometastasis (p < 0.001). The PET/CT plus MRI and HG model included the primary tumor SUVmax (p = 0.10) and histological grade (p = 0.079) regardless of their p values in the multivariate analysis, since they were considered to be clinically important information. The final PET/CT plus MRI and HG model demonstrated significantly better discrimination (C-statistic = 0.883) than that of the PET/CT and HG (C-statistic = 0.834) and the MRI and HG models (C-statistic = 0.813) with p-values <0.05. The weighted scores were assigned to each retained variable in the PET/CT plus MRI and HG model in **Table 3**.

**Table 2:** Predictors of axillary lymph node macrometastasis in the PET/CT and histological grade, MRI and histological grade, and PET/CT plus MRI and histological grade models and score weights of the predictive variables in the PET/CT plus MRI and histological grade model.

Predictors	Univariate analysis; p value	Multivariate analysis; β coefficients (SD), OR (95%CI), p value									
		The PET/CT and HG model			The MRI and HG model			The PET/CT plus MRI and HG model			
**Age** (≥ 57 vs. < 57 years)	0.73	β (SD)	OR (95%CI)	p value	β (SD)	OR (95%CI)	p value	β (SD)	OR (95%CI)	p value	Score
**Histological grade** (II and III vs. I)	0.003	0.98 (0.51)	2.66 (0.98–7.25)	0.055	1.20 (0.49)	3.33 (1.28–8.65)	0.013	0.95 (0.54)	2.59 (0.90–7.47)	0.079	3
**Nuclear grade** (2 and 3 vs. 1)	0.30										
**Ki-67 grade** (≥30% vs. <30%)	0.22										
**ER status** (Positive vs. Negative)	0.70										
**PgR status** (Positive vs. Negative)	0.36										
**HER2 status** (Positive vs. Negative)	0.22										
**Molecular subtype** (Non-luminal vs. Luminal)	0.78										
**Primary tumor SUVmax** (≥4.6 vs. <4.6)	< 0.001	1.30 (0.46)	3.69 (1.50–9.04)	0.004				0.82 (0.49)	2.26 (0.86–5.96)	0.099	2
**Lymph node SUVmax** (≥1.2 vs. <1.2)	< 0.001	2.71 (0.40)	15.0 (6.90–32.8)	<0.001				2.33 (0.43)	10.3 (4.41–23.9)	<0.001	7
**MRI tumor size** (≥19 vs. <19 mm)	< 0.001				1.51 (0.35)	4.55 (2.29–9.04)	< 0.001	1.16 (0.41)	3.19 (1.43–7.11)	0.005	3
**MRI lymph node size**											
**Long-axis diameter** (≥8 vs. <8 mm)	< 0.001										
**Cortical thickness** (≥5 vs. <5 mm)	< 0.001				1.56 (0.36)	4.77 (2.36–9.63)	< 0.001	1.01 (0.40)	2.74 (1.25–6.03)	0.012	3
C-statistic		0.834			0.813			0.883			

SD, standard deviation; OR, odds ratio; CI, confidence interval; HG, histological grade; β, β coefficients; ER, estrogen receptor; PgR, progesterone receptor; HER2, human epidermal growth factor receptor 2; MRI, magnetic resonance imaging.

**Table 3:** Determination of the cutoff score for axillary lymph node macrometastasis in the validation cohort.

Cutoff score	The proportion of low-risk patients	False negative rate	Sensitivity	Specificity	Positive predictive value	Negative predictive value
0	0% (0/74)	0% (0/9)	100% (9/9)	0% (0/65)	12% (9/74)	− (0/0)
1	12% (9/74)	0% (0/9)	100% (9/9)	14% (9/65)	14% (9/65)	100% (9/9)
2	12% (9/74)	0% (0/9)	100% (9/9)	14% (9/65)	14% (9/65)	100% (9/9)
3	19% (14/74)	0% (0/9)	100% (9/9)	20% (13/65)	15% (9/60)	100% (14/14)
4	41% (30/74)	11% (1/9)	89% (8/9)	45% (29/65)	18% (8/44)	97% (29/30)
5	41% (30/74)	11% (1/9)	89% (8/9)	45% (29/65)	18% (8/44)	97% (29/30)
6	61% (45/74)	11% (1/9)	89% (8/9)	68% (44/65)	28% (8/29)	98% (44/45)
7	65% (48/74)	22% (2/9)	78% (7/9)	71% (46/65)	27% (7/26)	96% (46/48)
8	65% (48/74)	22% (2/9)	78% (7/9)	71% (46/65)	27% (7/26)	96% (46/48)
9	78% (58/74)	33% (3/9)	67% (6/9)	85% (55/65)	38% (6/16)	95% (55/58)
10	80% (59/74)	44% (4/9)	56% (5/9)	85% (55/65)	33% (5/15)	93% (55/59)
11	82% (61/74)	44% (4/9)	56% (5/9)	88% (57/65)	38% (5/13)	93% (57/61)
12	86% (64/74)	44% (4/9)	56% (5/9)	92% (60/65)	50% (5/10)	94% (60/64)
13	88% (65/74)	44% (4/9)	56% (5/9)	94% (61/65)	56% (5/9)	94% (61/65)
14	89% (66/74)	44% (4/9)	56% (5/9)	95% (62/65)	56% (5/9)	94% (62/66)
15	89% (66/74)	44% (4/9)	56% (5/9)	95% (62/65)	56% (5/9)	94% (62/66)
16	93% (69/74)	67% (6/9)	33% (3/9)	97% (63/65)	60% (3/5)	91% (63/69)
17	93% (69/74)	67% (6/9)	33% (3/9)	97% (63/65)	60% (3/5)	91% (63/69)
18	93% (69/74)	67% (6/9)	33% (3/9)	97% (63/65)	60% (3/5)	91% (63/69)

**Figure 4:**
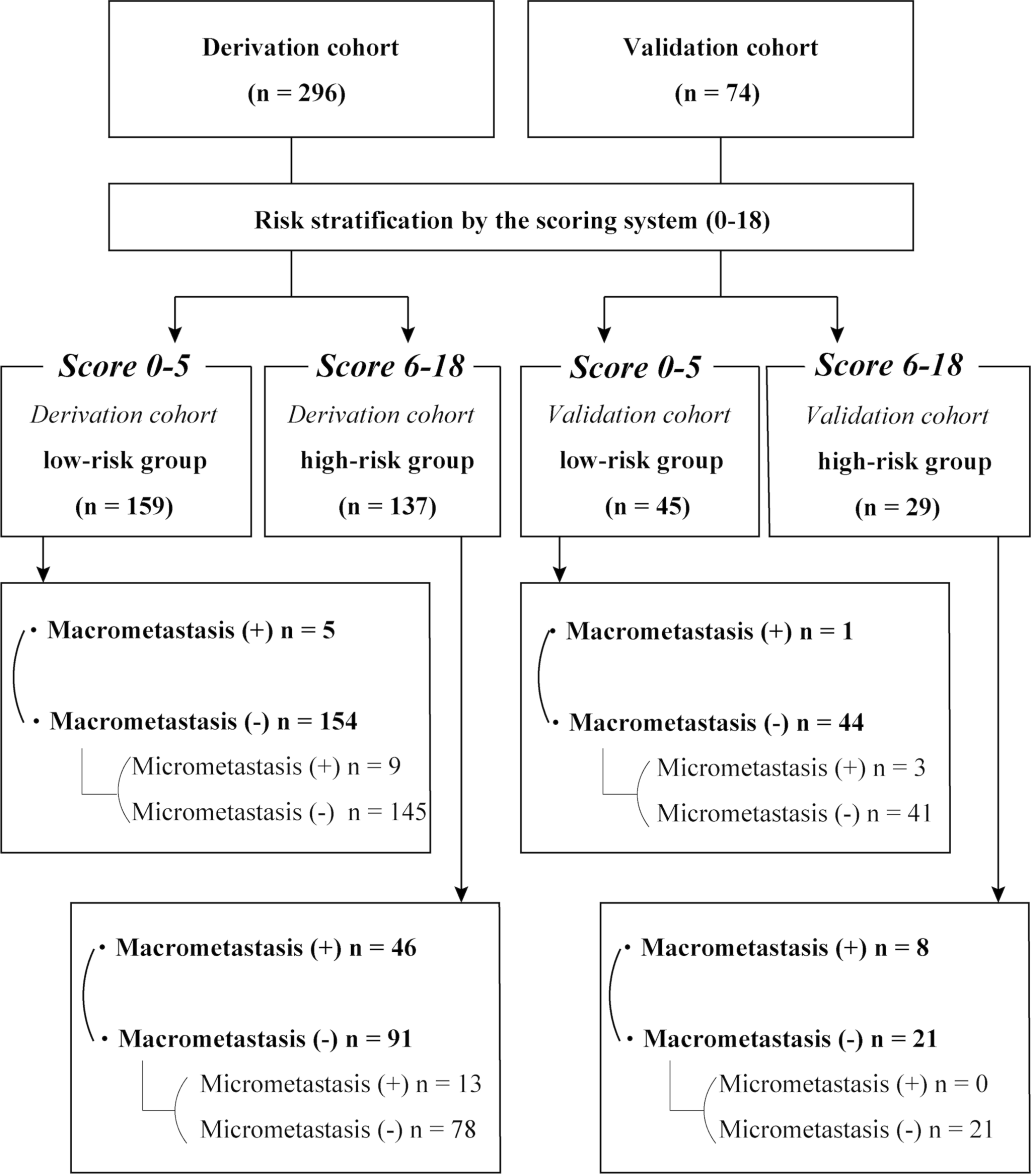
Results of the risk stratification by the scoring system in the derivation and validation cohorts.

**Figure 5:**
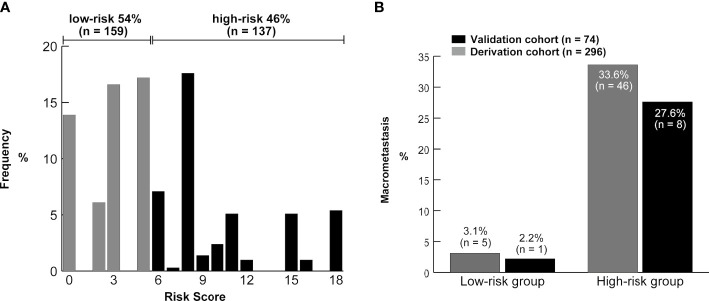
**(A)** Distribution of the risk scores from the derivation cohort. The brackets show the proportion of patients in the low-risk (0–5) and high-risk groups (≥6). **(B)** Frequency of the axillary lymph node macrometastasis according to the risk groups in the two cohorts.

We determined the optimal cutoff value to be 6; 54% of the patients were categorized into the low-risk group, with a false-negative rate of 11% in the derivation cohort. Using this simple integer scoring system, the total possible score was 18 points, and scores of 0–5 were classified as low risk and 6–18 were categorized as high risk (**Figure 4**). In the validation cohort, 61% (45/74) of patients were classified as low risk. The false-negative rate was 11% and a false-negative result was observed in only one patient (**Table 3**). **Figure 5** displays the frequency of the risk scores in the derivation cohort and the proportion of macrometastasis in the two cohorts. Additionally, a 5-fold cross-validation demonstrated an accuracy of 0.875. A nomogram based on the PET/CT plus MRI and HG model was developed to predict ALN macrometastasis, as shown in the **Supplementary Figure** (online).

### 3.4 Model construction for ALN micrometastasis

**Supplementary Table 2** (Supplementary Data) showed the patient characteristics between the two groups with (n = 25) or without ALN micrometastasis (n = 285). In univariate analysis, pSUVmax (≥7.4 vs. <7.4), MRI tumor size (≥14 mm vs. <14 mm), and Ki-67 (≥25% vs. <25%) demonstrated significant association with ALN micrometastasis (Supplementary Data, **Supplementary Table 3**). The risk prediction model combining pSUVmax with MRI tumor size showed an AUC of 0.668, and pSUVmax was the only independent prognostic factor for ALN micrometastasis (p = 0.046) identified by logistic regression analysis.

## 4 Discussion

This retrospective single-institutional study investigated the association between ALN macrometastasis and preoperative PET/CT, MRI, and biopsy findings, such as CNB or VAB. The clinical prediction model for ALN macrometastasis demonstrated significant discrimination compared to previously reported models utilizing either PET/CT or MRI with the findings of biopsy (19, 20). Therefore, we developed a simple integer risk score assessment to confirm its diagnostic accuracy for internal validation using a split-sample design, and the 5-fold cross-validation method was also applied to our prediction model. Additionally, a nomogram was created to predict ALN macrometastasis.

An improved PET/CT model has previously been reported; the model determined the optimal cutoff value of the pSUVmax for each molecular subtype (19). This may be biologically plausible since several reports have elucidated the association between HER2 oncogene expression and 18F-FDG uptake (21), and pSUVmax was negatively correlated with the ER and PR (22). In this study, the optimal cutoff value of pSUVmax for each subtype was established by applying the receiver-operating characteristic curve analysis (cutoff values: luminal A-like type, 4.6; luminal B-like type, 4.8; luminal-HER2 type, 5.9; pure HER2 type 13.5; and triple-negative type, 7.9). Similar to a previous study (23), in **Supplementary Table 4** (Supplementary Data), we demonstrated that primary tumor SUVmax of non-luminal type breast cancer was significantly higher than luminal type (p < 0.001); however, subtype-pSUVmax was not incorporated into the model eventually because subtype-PET/CT plus MRI model could not significantly improve its C-statistic as compared to that of the PET/CT plus MRI models (Supplementary Data, **Supplementary Table 5**). Our study assessed the metabolic data and morphologic details of the ALNs using contrast-enhanced MRI in a larger sample size.

The new diagnostic modality combining PET and MRI is PET/MRI (24); however, PET/MRI reportedly did not significantly improve the diagnostic performance for ALN metastasis compared to PET/CT and MRI (12). Partly owing to the poor cost-effectiveness and insufficient evidence, PET/MRI is not routinely indicated as well as PET/CT, especially for preoperative staging of early breast cancer, as mentioned in the National Comprehensive Cancer Network guideline 2022 (8). However, our findings may support the efficacy of a combination of PET/CT plus MRI for accurate axillary nodal staging and contain useful information for the PET/MRI interpretation. Moreover, the clinical benefits of PET/CT surveillance include the detection of distant metastasis, and regional lymph node involvement in the supraclavicular and internal mammary lymph node metastasis, which would be difficult to identify using other modalities. In fact, nine patients with primary breast cancer were diagnosed with internal mammary lymph node metastasis by PET/CT in our hospital between April 2017 and June 2022, which provided precise clinical upstaging and further treatment accessibility to patients with breast cancer involving internal mammary node lymph node chain, such as radiation therapy (25).

The PET/CT plus MRI and HG model demonstrated a low false-negative rate (11%) in the validation cohort, and only six patients with a median total score of 4 (range, 3–5) were misdiagnosed with node-negative breast cancer in the two cohorts. Considering the false negative rate of 7.5% (95% CI, 4.8–11.5%) of SLNB (26), this PET/CT plus MRI and HG model may not be inferior to SLNB for nodal staging. Every false-negative case had a single ALN macrometastasis, and they were all diagnosed as luminal type breast cancer. Four of six false negative cases were treated with lumpectomy followed by whole breast radiation therapy and adjuvant endocrine therapy. Thus, this model may seldom miss multiple ALN macrometastases and may be more compatible with patients with non-luminal breast cancer. Retrospectively, four of six false negative cases met the criteria of either MRI or PET/CT; two cases showed a cortical thickness of 5 mm, and three cases represented the SUVmax over 4.6.

Recent clinical prediction models utilizing the pretreatment radiomics features of contrast-enhanced MRI and contrast-enhanced CT also exhibited a high diagnostic accuracy for ALN metastasis in patients with breast cancer, with an AUC of approximately 0.900 in the validation cohort (27–29). In radiomics studies, prediction models are constructed using machine learning to improve the predictive ability, making it a promising method to analyze the morphological features of ALNs for better preoperative nodal staging prediction. In our study, clinicians treating breast cancer were traditionally familiar with the extracted imaging features, which are easy to measure. This simplicity is a clinically important advantage of this study because biopsy is recommended and performed routinely by breast surgeons in our country. The morphological evaluation of ALNs by MRI is highly informative not only for radiologists, but also for breast surgeons; therefore, we consider that our clinical prediction model using a simple measuring method may be helpful for preoperative nodal staging.

According to a randomized clinical trial (ACOSOG Z0011), no further axillary surgery was recommended by the National Comprehensive Cancer Network panel (24) and the 17th St. Gallen International Breast Cancer Consensus Conference in 2021 (30) for patients who underwent lumpectomy for T1 or T2 tumors with 1 to 2 positive SLNs, treated comprehensively with postoperative radiation and adjuvant drug therapy. The ACOSOG Z0011 trial revealed that the only one case of nodal recurrence was observed in the SLND alone group (n = 426). The unresected residual metastatic ALNs (in the patient’s body) after surgery may be locally controlled by adjuvant systemic therapy, radiation therapy, and the host immune system. However, clinical evidence about ALND omission for patients who have undergone total mastectomy is limited due to a relatively small number of participants compared to lumpectomy. The International Breast Cancer Study Group 23-01 trial conducted subgroup analysis to compare the breast-conserving surgery and total mastectomy groups, and found that the clinical outcome did not differ significantly between the two groups. Similarly, in the multicenter randomized ongoing clinical trial [SINODAR-ONE study (31)], started in 2015 in Italy for patients who underwent mastectomy, or lumpectomy plus radiation therapy for T1 or T2 tumors with 1 to 2 macrometastasis of SLNs, ALND did not improve disease-free survival and overall survival as compared to omitting ALND at 33–34 months median follow-up. The tumor burden in ALNs of patients in the SINODAR-ONE study was presumably higher than those in the ACOSOG Z0011 study since approximately half of the positive SLNs were micrometastases in the latter trial. Although the follow-up period is too short to confirm the prognosis, the results of SINODAR-ONE study may explain why the tumor burden of micrometastases rejected in our clinical prediction model is negligible.

Contrastingly, the RxPONDER trial (9) used the 21-gene breast cancer assay to predict the benefits of adjuvant chemotherapy and reported benefits for premenopausal women with ALN micrometastasis in 2-year landmark invasive disease-free survival. In our analysis, pSUVmax was the only independent prognostic factor for ALN micrometastasis. PET/CT could be a potential imaging modality to detect ALN micrometastasis; however, only 3 of the 25 patients with ALN micrometastasis in the two cohorts showed FDG accumulation in ALNs. All of the 3 patients with false-negative cases of ALN micrometastasis in the validation cohort met the criteria for pSUVmax, whereas the cortical thickness of the lymph node exceeded 5 mm in one patient. This is biologically plausible, since ALN micrometastatic foci may not cause an obvious morphological change of the cortex structure of lymph nodes as compared to ALN macrometastasis. We suggest that future studies to establish the clinical prediction model for ALN micrometastasis using 18F-FDG PET/CT should focus on the pSUVmax.

Another randomized clinical trial [EORTC 10981-22023 AMAROS trial (32)] demonstrated that axillary radiotherapy following total mastectomy for T1 or T2 primary breast cancer was not inferior to ALND in terms of the local recurrence rate. The main purpose of ALND has been to reduce the risk of local recurrence; however, it is losing its importance owing to the advances in adjuvant axillary radiotherapy and systemic therapy. Moreover, several clinical trials eliminating SLNB were launched, not only for low-risk elderly patients followed by adjuvant endocrine therapy (33, 34), but also for younger patients (35) including clinically node-negative patients following neoadjuvant chemotherapy (36). To eliminate the SLNB procedure, a population-based analysis of the suitable risk stratification was performed for elderly patients with breast cancer (37). We consider that the PET/CT plus MRI and HG model in this study may improve the preoperative risk evaluation of ALN macrometastasis. The low specificity and low positive predictive values are weaknesses of the present study; however, each of the 204 (55%) of 370 patients, who were retrospectively categorized into the low-risk group, routinely underwent invasive axillary surgery in the clinical setting. In fact, of the 204 patients in the low-risk group, only six (2.9%) were node positive, all of whom had luminal-type breast cancer with a single macrometastais, and received adjuvant endocrine therapy. Our prediction model for ALN macrometastasis may be useful in the future to consider the possibility of safely omitting SLNB for such a low-risk group, although it requires rigorous external validation and confirmation using a prospective clinical trial.

This study has several limitations. First, because the primary breast lesions and ALNs were relatively small, the partial-volume effect of PET/CT could have resulted in the underestimation of the SUVmax (38). Second, a history of neoadjuvant chemotherapy made pathological evaluation for the presence of lymph node metastasis challenging; therefore, the number of HER2-positive type and triple-negative type breast cancers were limited by neoadjuvant chemotherapy, potentially creating a selection bias. Third, we had to exclude invasive lobular carcinomas, which occupy 10–15% of the breast cancer from this study because they showed lower conspicuity on PET/CT as compared to IDC (39). Fourth, this retrospective analysis was based on a single-center database. We performed an internal validation in a split-sample design and a 5-fold cross validation; however, an additional multicenter controlled prospective evaluation should be performed to confirm the clinical significance of this simple integer score for the ALN macrometastasis. Fifth, the number of the patients who had micrometastasis was small; therefore, the results are susceptible to overfitting. Currently, PET/CT and MRI would require careful interpretation and prospective assessments, since vaccine-induced adenopathy and FDG accumulation in ALNs may occur following coronavirus disease 2019 vaccination (40). In Japan, SLNB, a contrast-enhanced MRI, and a PET/CT scan cost approximately $400, $170, and $750, respectively. Although the expenditures on PET/CT scans seemed to be generally lower than those in other countries, a cost-effective analysis is necessary for greater application of this radiologic imaging strategy in the clinical practice (41).

In conclusion, our clinical prediction model utilizing PET/CT plus MRI demonstrated significantly improved diagnostic accuracy over previously reported models based on either PET/CT or MRI. These results provided potentially useful information for PET/MRI interpretation, and may support the clinical efficacy of PET/MRI. It is necessary that we perform further internal and external validation of the preoperative risk assessment using our integrated model in considering individualized therapy for patients with IDC.

## Data availability statement

Data analyzed during the study are available from the corresponding author by request. Requests to access these datasets should be directed to Shun Kawaguchi, kshun51@gmail.com.

## Ethics statement

The study is exempt from informed patient consent and board approval by the Toranomon Hospital Ethics Board. The ethics committee waived the requirement of written informed consent for participation.

## Author contributions

Conception/design of the work: HK and SK. Acquisition of the data: SK, NT, and HK. Analysis of the data: SK, NT, and HK. Interpretation of the data: SK, HK, MI, MS, JS, KK, KT, YK, and NT. Drafting the work: SK, HK, and NT. All authors revised the manuscript, approved the final version and agreed to be fully accountable for all aspects of the work.

## Funding

Funding was provided by Okinaka Memorial Institute for Medical Research.

## Acknowledgments

We thank the Institute for Education and Research in Clinical Epidemiology for their valuable guidance in performing the statistical analysis and the review and suggestions for its results. We express our sincere thanks to Dr. Naoya Gomi (radiologist, Department of Diagnostic Imaging, Cancer Institute Hospital, Japanese Foundation of Cancer Research) for helpful discussions and comments on the manuscript and figures. We are also grateful to Kei Fukuzawa and Kazuaki Mori (radiation technologist, Toranomon Hospital) for providing valuable information of the device configuration of MRI and PET/CT.

## Conflict of interest

The authors declare that the research was conducted in the absence of any commercial or financial relationships that could be construed as a potential conflict of interest.

## Publisher’s note

All claims expressed in this article are solely those of the authors and do not necessarily represent those of their affiliated organizations, or those of the publisher, the editors and the reviewers. Any product that may be evaluated in this article, or claim that may be made by its manufacturer, is not guaranteed or endorsed by the publisher.
