# Supplementary material for: Clinical prediction model based on 18F-FDG PET/CT plus contrast-enhanced MRI for axillary lymph node macrometastasis
Source: Front Oncol. 2022 Sep 13;12:989650. doi: 10.3389/fonc.2022.989650 (PMC9513385; doi:10.3389/fonc.2022.989650)
Supplement: Supplementary file 1 [file DataSheet_1.docx]

Supplementary Material

# Supplementary Data

## Detailed 18F-fluoro-2-deoxy-D-glucose PET/CT Protocol

The PET/CT scanning protocol was as follows. The patients fasted for at least 6 h before intravenous administration of approximately 4 MBq/kg of 18F-fluoro-2-deoxy-D-glucose (maximum, 300 MBq) via an indwelling catheter. Serum glucose levels were determined, and patients with blood glucose levels <200 mg/dL were selected for the procedure. A whole-body scan starting approximately 90 min after intravenous administration was acquired from the skull base to the upper thighs with patients in the supine position. All images were reconstructed with a 2-mm slice thickness using an iterative reconstruction algorithm.

## Detailed Contrast-enhanced MRI Protocol And Image Analysis

The MRI scanning protocol was as follows. Patients were imaged in the prone position. T1-weighted imaging (repetition time/echo time, 640/10 ms; phase encoding direction, right-to-left; flip angle, 90º; field of view, 320 × 320 mm, thickness, 3 mm; matrix, 304 × 384), T2-weighted imaging, and fat-suppressed imaging (repetition time/echo time, 10700/80 ms; phase encoding direction, right-to-left; flip angle, 80º; field of view, 320 × 320 mm, thickness, 3 mm; matrix, 288 × 288) were obtained as precontrast imaging. After intravenous administration of gadoteridol (Prohance, Bracco, Milan, Italy) using a single dose of 0.2 mmol/kg body weight at a flow rate of 3 mL/s, dynamic contrast-enhanced, fat-suppressed, gradient echo, T1-weighted axial imaging (repetition time/echo time, 3.8/1.95 ms; phase encoding direction, right-to-left; flip angle, 12º; field of view, 320 × 320 mm; thickness, 2 mm; matrix, 352 × 384) was performed. The time interval between sequential acquisitions of three images was 60 s. Breast and axillary MR images with breast cancer were retrospectively reviewed, and the readers were blinded to the clinical information regarding ALN analysis during interpretation of the radiogram. Image analysis was performed separately by two readers, a breast surgeon (S.K.) and an experienced breast surgeon (H.K.) with 5 and >20 years’ experience in breast imaging, respectively. The readers were unaware of each other’s findings until the completion of their interpretation. A consensus was reached with discussion in case of disagreement between the readings. The long diameter of the primary breast cancer was calculated in both the sagittal and horizontal dimensions, and the highest value was recorded.

## Detailed Pathological Evaluation

All reagents were prediluted (Hoffmann-La Roche, Basel, Switzerland). ER and PR were scored as positive or negative, with a nuclear immunostaining cut-off of 1%. HER2 positivity was defined as an immunohistochemical staining score of 3+; when weak to moderate complete membrane staining was observed in more than 10% of the tumor cells, its status depended on dual-probe in situ hybridization analysis demonstrating HER2 gene amplification according to recommendations of the 2018 American Society of Clinical Oncology and College of American Pathologists guidelines.^15^

Based on the different combinations of ER, PR, HER2, and Ki-67 expression, the molecular subtypes were categorized into five subgroups in the analysis:

1. Luminal A-like type: ER (+), PR (+), HER2 (-), and Ki-67 low (<20%)

2. Luminal B-like type: ER (+) and/or PR (+), HER2 (-), and Ki-67 high (≥20%)

3. Luminal-HER2 type: ER (+) and/or PR (+), HER2 (+)

4. Pure HER2 type: ER (-), PR (-), and HER2 (+)

5. Triple-negative type: ER (-), PR (-), and HER2 (-)

# Supplementary Figure


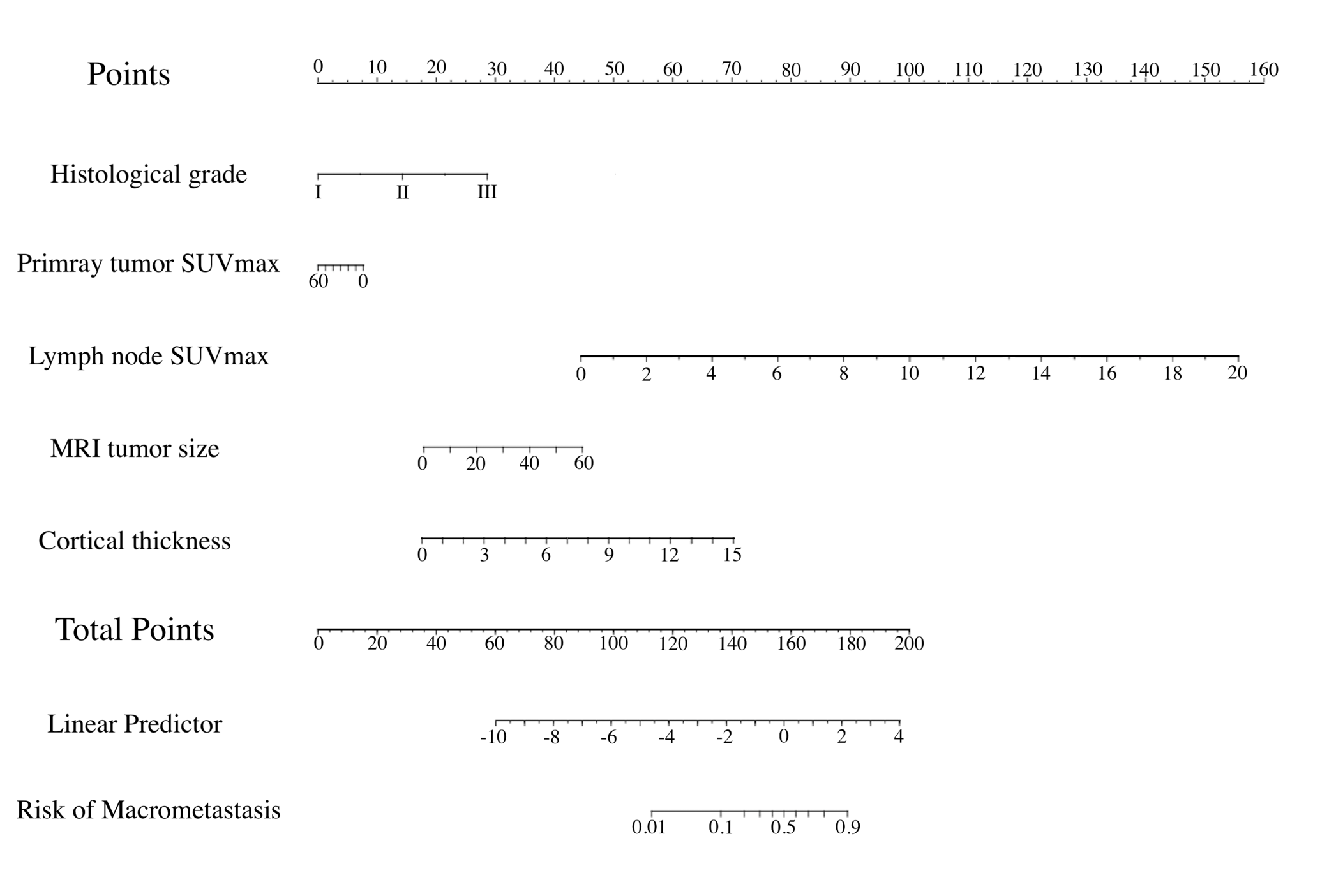


# Supplementary Tables

**Supplementary Table 1.** Comparison of patients' characteristics between the derivation and validation cohorts

| Characteristics | Derivation cohort  (n = 296) | Validation cohort  (n = 74) | p value |  |
| --- | --- | --- | --- | --- |
| Age, years* | 55.8 ± 12.1 | 57.5 ± 13.9 | 0.29 |  |
| Histological grade |  |  | 0.67 |  |
| I | 96 (32) | 20 (27) |  |  |
| II | 171 (58) | 47 (64) |  |  |
| III | 29 (10) | 7 (9) |  |  |
| Nuclear grade |  |  | 0.93 |  |
| 1 | 153 (52) | 37 (50) |  |  |
| 2 | 110 (37) | 28 (38) |  |  |
| 3 | 33 (11) | 9 (12) |  |  |
| Ki-67 grade |  |  | 0.20 |  |
| <20% | 157 (53) | 33 (45) |  |  |
| ≥20% | 139 (47) | 41 (55) |  |  |
| Lymphovascular invasion |  |  | 0.58 |  |
| Absence | 200 (68) | 53 (72) |  |  |
| Presence | 96 (32) | 21 (28) |  |  |
| Tumor size in pathology* (mm) | 13.8 ± 8.3 | 14.0 ± 10.2 | 0.84 |  |
| Lymph node macrometastasis |  |  | 0.38 |  |
| Absence | 245 (83) | 65 (88) |  |  |
| Presence | 51 (17) | 9 (12) |  |  |
| Molecular subtypes |  |  | 0.45 |  |
| Luminal A-like (Ki-67<20%) | 135 (46) | 30 (41) |  |  |
| Luminal B-like (Ki-67≥20%) | 114 (39) | 28 (38) |  |  |
| Luminal-HER2 | 21 (7) | 6 (8) |  |  |
| Pure HER2 | 11 (4) | 2 (3) |  |  |
| Triple-negative | 15 (5) | 8 (11) |  |  |
| Nodal FDG uptake finding |  |  | 0.86 |  |
| Negative | 246 (83) | 61 (82) |  |  |
| Positive | 50 (17) | 13 (18) |  |  |
| Primary tumor SUVmax* | 7.2 ± 6.3 | 8.0 ± 8.7 | 0.38 |  |
| Lymph node SUVmax* | 4.1 ± 4.0 | 5.7 ± 7.0 | 0.29 |  |
| MRI tumor size* (mm) | 16.3 ± 8.8 | 16.2 ± 9.0 | 0.95 |  |
| MRI lymph node size* (mm) |  |  |  |  |
| Long-axis diameter | 7.7 ± 3.7 | 8.2 ± 3.4 | 0.31 |  |
| Cortical thickness | 4.4 ± 2.2 | 4.2 ± 2.0 | 0.51 |  |

Note. HER2, human epidermal growth factor receptor 2; FDG, 18F-fluoro-2-deoxy-D-glucose; SUVmax, maximum standardized uptake value; MRI, magnetic resonance imaging; SLNB, sentinel lymph node biopsy; ALND, axillary lymph node dissection

* Data are means ± standard deviation

**Supplementary Table 2.** Patients' characteristics with or without axillary lymph node micrometastasis

| Characteristics | Micrometastasis (-) Macrometastasis (-)  (n = 285) | Micrometastasis (+) Macrometastasis (-)  (n = 25) | p value |
| --- | --- | --- | --- |
| Age, years* | 56.5 ± 12.5 | 52.1 ± 11.9 | 0.10 |
| Histological grade |  |  | 1 |
| I | 101 (35) | 9 (36) |  |
| II | 156 (55) | 14 (56) |  |
| III | 28 (10) | 2 (8) |  |
| Nuclear grade |  |  | 0.19 |
| 1 | 154 (54) | 9 (36) |  |
| 2 | 99 (35) | 13 (52) |  |
| 3 | 32 (11) | 3 (12) |  |
| Ki-67 grade |  |  | 0.058 |
| <20% | 153 (54) | 8 (32) |  |
| ≥20% | 132 (46) | 17 (68) |  |
| Lymphovascular invasion |  |  | <0.001 |
| Absence | 240 (84) | 3 (12) |  |
| Presence | 45 (16) | 22 (88) |  |
| Tumor size in pathology* (mm) | 12.3 ± 7.2 | 15.4 ± 6.6 | 0.024 |
| Molecular subtypes |  |  | 0.039 |
| Luminal A-like (Ki-67 < 20%) | 135 (47) | 8 (32) |  |
| Luminal B-like (Ki-67 ≥ 20%) | 103 (36) | 13 (52) |  |
| Luminal-HER2 | 20 (7) | 0 (0) |  |
| Pure HER2 | 7 (2) | 3 (12) |  |
| Triple-negative | 20 (7) | 1 (4) |  |
| Nodal FDG uptake finding |  |  | 0.48 |
| Negative | 260 (91) | 22 (88) |  |
| Positive | 25 (9) | 3 (12) |  |
| Primary tumor SUVmax* | 6.8 ± 6.2 | 8.6 ± 5.8 | 0.15 |
| Lymph node SUVmax* | 2.4 ± 2.0 | 2.8 ± 0.6 | 0.47 |
| MRI tumor size* (mm) | 14.5 ± 6.9 | 20.3 ± 12.2 | <0.001 |
| MRI lymph node size* (mm) |  |  |  |
| Long-axis diameter | 7.2 ± 3.0 | 7.9 ± 3.7 | 0.27 |
| Cortical thickness | 3.9 ± 1.5 | 4.4 ± 1.5 | 0.067 |

Note. HER2, human epidermal growth factor receptor 2; FDG, 18F-fluoro-2-deoxy-D-glucose; SUVmax, maximum standardized uptake value; MRI, magnetic resonance imaging; SLNB, sentinel lymph node biopsy; ALND, axillary lymph node dissection

* Data are means ± standard deviation

**Supplementary Table 3.** Predictors of axillary lymph node micrometastasis

| Predictors | Univariate analysis; p value | Multivariate analysis;  β coefficients (SD), OR (95% CI), p value | | |
| --- | --- | --- | --- | --- |
|  |  | The PET/CT plus MRI model | | |
|  | | β coefficients  (SD) | OR  (95% CI) | p value |
| **Ki-67 grade**  (≥25% vs. <25%) | 0.017 |  |  |  |
| **Primary tumor SUVmax**  (≥7.4 vs. <7.4) | 0.006 | 0.96  (0.48–2.00) | 2.61  (1.02–6.70) | 0.046 |
| **MRI tumor size**  (≥14 vs. <14 mm) | 0.022 | 0.59  (0.52–1.14) | 1.80  (0.65–4.99) | 0.26 |
| **MRI lymph node size** |  |  |  |  |
| **Cortical thickness**  (≥4 vs. <4 mm) | 0.10 |  |  |  |
| C-statistic |  | 0.668 | | |

Note. SD, standard deviation; OR, odds ratio; CI, confidence interval; ER, estrogen receptor; PgR, progesterone receptor; HER2, human epidermal growth factor receptor 2; MRI, magnetic resonance imaging

**Supplementary Table 4.** Comparison of the molecular subtypes of breast cancer

| Characteristics | Luminal A-like  (Ki-67 <20%) (n = 165) | Luminal B-like (Ki-67 ≥20%) (n = 142) | Luminal-HER2  (n = 27) | Pure  HER2  (n = 13) | Triple- negative  (n = 23) | p value |
| --- | --- | --- | --- | --- | --- | --- |
| Age, years* | 56.9 ± 12.2 | 55.0 ± 12.3 | 55.1 ± 11.8 | 59.5 ± 11.0 | 57.2 ± 15.5 | 0.56 |
| Histological grade |  |  |  |  |  | <0.001 |
| I | 78 (47) | 33 (23) | 4 (15) | 0 (0) | 1 (4) |  |
| II | 87 (53) | 96 (68) | 19 (70) | 6 (46) | 10 (43) |  |
| III | 0 (0) | 13 (9) | 4 (15) | 7 (54) | 12 (52) |  |
| Nuclear grade |  |  |  |  |  | <0.001 |
| 1 | 143 (87) | 37 (26) | 7 (26) | 0 (0) | 3 (13) |  |
| 2 | 22 (13) | 91 (64) | 15 (56) | 6 (46) | 4 (17) |  |
| 3 | 0 (0) | 14 (10) | 5 (19) | 7 (54) | 16 (70) |  |
| Ki-67 grade |  |  |  |  |  | <0.001 |
| <20% | 165 (100) | 0 (0) | 5 (19) | 0 (0) | 3 (13) |  |
| ≥20% | 0 (0) | 142 (100) | 22 (81) | 13 (100) | 20 (87) |  |
| Lymphovascular invasion |  |  |  |  |  | 0.043 |
| Absence | 125 (76) | 88 (62) | 16 (59) | 7 (54) | 17 (74) |  |
| Presence | 40 (24) | 54 (38) | 11 (41) | 6 (46) | 6 (26) |  |
| Tumor size* (mm) | 11.2 ± 7.8 | 15.8 ± 9.0 | 16.3 ± 9.8 | 14.6 ± 6.0 | 16.6 ± 7.7 | <0.001 |
| Nodal FDG uptake finding |  |  |  |  |  | 0.006 |
| Negative | 144 (87) | 121 (85) | 18 (67) | 9 (69) | 15 (65) |  |
| Positive | 21 (13) | 21 (15) | 9 (33) | 4 (31) | 8 (35) |  |
| Primary tumor SUVmax* | 5.0 ± 4.4 | 8.7 ± 8.0 | 8.7 ± 5.9 | 10.6 ± 6.3 | 12.6 ± 8.8 | <0.001 |
| Lymph node SUVmax* | 3.7 ± 3.4 | 4.5 ± 3.9 | 5.8 ± 7.8 | 4.8 ± 3.4 | 4.6 ± 6.1 | 0.89 |
| MRI tumor size* (mm) | 13.9 ± 7.7 | 17.7 ± 9.3 | 19.7 ± 10.1 | 17.0 ± 6.7 | 19.5 ± 8.8 | <0.001 |
| MRI lymph node size* |  |  |  |  |  |  |
| Long-axis diameter (mm) | 7.3 ± 3.3 | 7.9 ± 4.1 | 8.4 ± 2.4 | 10.5 ± 5.0 | 8.5 ± 2.7 | 0.022 |
| Cortical thickness (mm) | 4.0 ± 1.8 | 4.5 ± 2.6 | 4.7 ± 2.1 | 4.8 ± 2.1 | 4.6 ± 1.9 | 0.26 |

Note. HER2, human epidermal growth factor receptor 2; FDG, 18F-fluoro-2-deoxy-D-glucose; SUVmax, maximum standardized uptake value; MRI, magnetic resonance imaging

* Data are means ± standard deviation

**Supplementary Table 5.** Predictors of axillary lymph node macrometastasis utilizing the subtype- SUVmax of primary tumor

| Predictors | Univariate-  analysis;  p value | Multivariate analysis;  β coefficients (SD), OR (95% CI), p value | | | |  |  |
| --- | --- | --- | --- | --- | --- | --- | --- |
|  |  | The histological grade and subtype-PET/CT model | | | The histological grade and subtype-PET/CT plus MRI model | | |
| **Age, years**  (≥57 vs. <57) | 0.12 | β  (SD) | OR  (95% CI) | p value | β  (SD) | OR  (95% CI) | p value |
| **Histological grade**  (II and III vs. I) | <0.001 | 1.19  (0.49) | 3.30  (1.27–8.57) | 0.014 | 1.20  (0.52) | 3.31  (1.20–9.1-) | 0.021 |
| **Nuclear grade**  (2 and 3 vs. 1) | 0.32 |  |  |  |  |  |  |
| **Ki-67 grade**  (≥30% vs. <30%) | 0.46 |  |  |  |  |  |  |
| **ER status**  (Positive vs. Negative) | 1 |  |  |  |  |  |  |
| **PgR status**  (Positive vs. Negative) | 0.48 |  |  |  |  |  |  |
| **HER2 status**  (Positive vs. Negative) | 0.12 |  |  |  |  |  |  |
| **Molecular subtype**  (Non-luminal vs. Luminal) | 0.82 |  |  |  |  |  |  |
| **Primary tumor SUVmax**  (≥4.6 vs. <4.6) | <0.001 |  |  |  |  |  |  |
| **Subtype-Primary tumor SUVmax** | <0.001 | 1.08  (0.38) | 2.94  (1.41–6.14) | 0.004 | 0.57  (0.41) | 1.78  (0.79–3.97) | 0.16 |
| **Lymph node SUVmax**  (≥1.2 vs. <1.2) | <0.001 | 2.56  (0.35) | 13.0  (6.54–25.8) | <0.001 | 2.10  (0.38) | 8.18  (3.90–17.2) | <0.001 |
| **MRI tumor size**  (≥19 vs. < 19 mm) | <0.001 |  |  |  | 1.02  (0.37) | 2.78  (1.34–5.77) | 0.006 |
| **MRI lymph node size** |  |  |  |  |  |  |  |
| **Long-axis diameter**  (≥8 vs. < 8 mm) | <0.001 |  |  |  |  |  |  |
| **Cortical thickness**  (≥5 vs. < 5 mm) | <0.001 |  |  |  | 1.15  (0.36) | 3.15  (1.54–6.43) | 0.002 |
| C-statistic |  | 0.825 | | | 0.875 | | |

Note. SD, standard deviation; OR, odds ratio; CI, confidence interval; β, β coefficients; ER, estrogen receptor; PgR, progesterone receptor; HER2, human epidermal growth factor receptor 2; MRI, magnetic resonance imaging
